# In-Cell NMR: Analysis of Protein–Small Molecule Interactions, Metabolic Processes, and Protein Phosphorylation

**DOI:** 10.3390/ijms20020378

**Published:** 2019-01-17

**Authors:** Amit Kumar, Lars T. Kuhn, Jochen Balbach

**Affiliations:** 1 Astbury Centre for Structural Molecular Biology, School of Molecular and Cellular Biology, University of Leeds, Leeds LS2 9JT, UK; 2 Institute of Physics, Biophysics, Martin–Luther–University Halle–Wittenberg, 06120 Halle, Germany; 3 Department of Diabetes, Faculty of Lifesciences and Medicine, King’s College London, Great Maze Pond, London SE1 1UL, UK; 4 Centre for Structure und Dynamics of Proteins (MZP), Martin–Luther–University Halle–Wittenberg, 06120 Halle, Germany

**Keywords:** protein NMR, in-cell NMR, in-situ NMR, DNP, review

## Abstract

Nuclear magnetic resonance (NMR) spectroscopy enables the non-invasive observation of biochemical processes, in living cells, at comparably high spectral and temporal resolution. Preferably, means of increasing the detection limit of this powerful analytical method need to be applied when observing cellular processes under physiological conditions, due to the low sensitivity inherent to the technique. In this review, a brief introduction to in-cell NMR, protein–small molecule interactions, posttranslational phosphorylation, and hyperpolarization NMR methods, used for the study of metabolites in cellulo, are presented. Recent examples of method development in all three fields are conceptually highlighted, and an outlook into future perspectives of this emerging area of NMR research is given.

## 1. General Introduction to In-Cell NMR

Most biological pathways are controlled by macromolecules. In order to study the structure and function of biomolecules, in vitro studies are usually applied; then, the resulting data are extrapolated to the native cellular environment. Although such approaches provide a wealth of information surrounding the structure–function activity of biomolecules, they lack the context of a native-complex environment [1,2]. The function of molecules in vivo may differ from that determined in vitro because their native network of interactions within the cell is missing.

**Figure 1:**
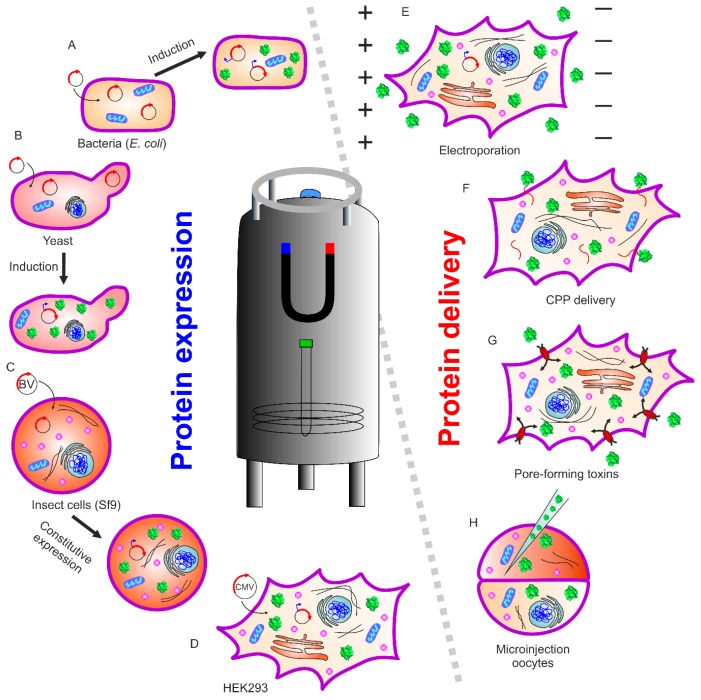
Schematic overview of different known approaches in in-cell NMR. (Left) Endogenously expressed and isotopically labeled protein can be achieved by transferring the expression vector containing the gene of interest into (**A**) bacteria, (**B**) yeast, (**C**) insect cell lines, and (**D**) mammalian cells. (Right) An alternate way of in-cell NMR, where isotopically labeled protein is exogenously prepared followed by delivery into eukaryotic cells with different methods such as (**E**) electroporation, (**F**) attaching protein with cell-penetrating peptides (CPP), (**G**) protein transport via pore-forming toxins, and (**H**) microinjection-mediated delivery into *Xenopus leavis* oocytes.

In-cell NMR spectroscopy provides a direct readout of protein–protein and ligand–protein interactions in the cellular environment. Therefore, this method gains added value to in vitro based techniques such as cryo-electron microscopy, X-ray crystallography, and hydrogen–deuterium exchange mass spectrometry, generating a wealth of information such as changes in structure and/or dynamics between the free and bound forms. However, these in vitro methods may not fully reflect the protein state in vivo, as the experimental conditions and protein constructs are optimized to obtain the best resolution with each respective method. Electron microscopy provides cellular structural features, but physiological temperatures and molecule sizes below 50 kDa are still challenging for high-resolution studies [3]. In-cell NMR spectroscopy is an ideal technique to study (at atomic resolution) the structural features of biomolecules, their function, and their interactions while they remain in their native cellular environment, as reviewed recently (e.g., References [1,2,4,5]). This technique is non-invasive and provides structural and biochemical details of macromolecules in solution, while applied in living cells over a wide range of parameters including temperature and pH. NMR methods are ensemble methods, meaning that the outcome is sample-averaged information originating from various molecules in many cells. Thus, the information obtained reflects the global properties of molecules without sub-cellular resolution. Historically, in vivo NMR started with studies of small molecules within living organisms/cells. One of the first in-cell NMR approaches to obtain high-resolution information of biomacromolecules (e.g., proteins) was described by Serber et al. [6,7] inside living cells (Figure 1). In their study, the authors chose two globular soluble proteins, the N-terminal domain of bacterial mercuric ion reductase (Nmer) A and human calmodulin, to explore protein in-cell NMR. In their approach, they utilized conventional recombinant protein expression and isotope labeling in bacterial cells, while considering other parameters such as cell growth, induction time, cell viability, isotopic labeling type, and NMR line broadening. Expression was optimized to obtain sufficient signal, much above that of other cellular proteins, to be detected by NMR spectroscopy.

Different types of isotope labeling of protein samples for in-cell NMR are available. Uniform ^15^N labeling was found to be most useful and the first choice for most of the studies (Figure 1a,b). The higher natural abundance of ^13^C in biomolecules, compared to ^15^N, renders this carbon isotope as the sole modification unsuitable for in-cell NMR studies. An alternative approach to uniform ^13^C enrichment is the specific labeling of amino acids [7]. Here, methyl-^13^C methionine labeling was a successful strategy to detect side-chain carbons well above the cellular background [8]. Yet another approach is the incorporation of non-natural amino acids containing ^19^F. This approach turned out to be a feasible means of investigating protein dynamics in the cellular environment. The advantage of ^19^F-labeled protein is that the in-cell NMR spectrum is virtually free of background [9,10]. 

Further developments of in-cell NMR led to methods such as structure interactions NMR (STINT-NMR), cross-correlated relaxation-induced polarization transfer NMR (CRIPT-NMR), and small-molecule interactor libraries NMR (SMILI-NMR). STINT-NMR allowed the study of protein–protein interactions while two molecules are heterologously overexpressed at different time points inside the same bacteria. Firstly, the ^1^H–^15^N HSQC spectrum of the ^15^N-labeled protein of interest is recorded within the cellular environment. Following this, the ^15^N growth medium is exchanged with an unlabeled medium to overexpress the interaction partner inside the cell. The changes in the chemical environment of the ^15^N nuclei are observed with time as the concentration of unlabeled binding partner increases. Burz et al. first demonstrated STINT-NMR applications by studying the interaction between a ubiquitin-binding peptide and the signal transducing adaptor molecule 2 protein (STAM2) [11,12]. Subsequently, STINT-NMR was applied to study the interactions between prokaryotic ubiquitin-like protein Pup-GGQ, mycobacterial proteasomal ATPase, Mpa, and the Mtb proteasome core particle (CP). These studies addressed the question of transient binding of Mpa to the proteasome CP that eventually controls the fate of Pup [13]. CRIPT-NMR is yet another in-cell NMR method that allows the identification of interacting surfaces presented on target ^15^N-labeled proteins within eukaryotic cells, such as HeLa [14]. High-molecular-weight protein molecules can be studied in cells using relaxation optimized ^15^N-edited cross-relaxation enhanced polarization transfer (CRINEPT), heteronuclear multiple quantum coherence (HMQC), transverse relaxation optimized spectroscopy (TROSY) (^1^H-^15^N CRINEPT–HMQC–TROSY) experiments. This method is advantageous due to its relative insensitivity to unavoidable magnetic field inhomogeneity and its high sensitivity to NMR signals. In the in-cell NMR experiment, proton relaxation was minimized by exchanging α and β protons of the amino acids for deuterons called reduced proton density (REDPRO) labeling. Thereafter, a calibration of the CRINEPT transfer time is required to achieve maximum in-cell NMR peak intensities. The in-cell NMR spectrum of the fully expressed protein is compared with its in vitro spectrum and its spectrum in cell lysate. Thus, the interacting surfaces are mapped based upon the residues exhibiting the greatest change in peaks position/intensity. SMILI-NMR was developed, by the same authors, to follow the interactions of proteins with small molecules by in-cell NMR. This technique relies on complex formation of isotope-labeled proteins with small molecules to screen in cellulo entire libraries. The protein of interest gets uniformly labeled with NMR-active heteronuclei under in-cell NMR conditions. This is followed by addition of cell-penetrable small molecules. Monitoring in-cell NMR protein spectra, thus, allows direct observation of protein–small molecule complex formation, in addition to any possible conformational changes [15].

The comprehensive in-cell NMR methods described above to reveal protein–protein or protein–small molecule interactions could potentially act as a bridge between structural and cellular biology. These techniques, already providing excellent results within bacterial systems, unleashed their full potential when applied to eukaryotic and mammalian cell systems. Yeast expression systems provide a simple platform for the study of eukaryotic protein molecules (Figure 1b). This system has the advantage of a unicellular organism with an established expression system and supplement control. The study of proteins within different cellular compartments can be readily performed in yeast [16]. Although the yeast expression system is quite valuable, it suffers from the short lifetime of cells in the NMR sample tube, limiting the experimental observation of events to just a few hours. To overcome this limitation, micro-bioreactors are available for both bacteria/yeast and human cells, which can supply fresh medium and air, and maintain a stable pH value [17,18].

In-cell NMR was first performed in eukaryotic cells on the *Xenopus laevis* oocyte cell system (Figure 1H) [19,20,21]. This was achieved by preparing protein, injecting into the oocytes, resulting in high labeling selectivity and almost no cellular background. This method proved to be an excellent tool to study posttranslational protein modifications. The Selenko group and others studied serine, threonine, or tyrosine phosphorylation in physiological environments using reconstituted kinase reactions, cell extracts, and intact cells [22]. Real-time monitoring provides additional information about mechanistic insights into modification hierarchies. These may include inhibitory [23], sequential [24], stimulatory [25,26], or “priming” events in phosphorylation cascades. The stepwise modifications of adjacent casein kinase 2 binding sites in the SV40 large T antigen regulatory region, coupled to intermediate substrate release, was disclosed using in situ NMR within *Xenopus laevis* egg extracts and whole live oocyte cells [26]. 

Inomata et al. applied in-cell NMR spectroscopy in cultured human cells utilizing the cell-penetrating peptide (CPP)2 derived from the human immunodeficiency virus (HIV-1) trans-activator of transcription (Tat) protein. The method relies upon the fusion of (CPP)2 to the protein of interest for internalization into cells (Figure 1F). It is also possible to covalently link (CPP)2 to the protein of interest via a disulfide bond. Such a linkage is cleaved upon internalization in the reducing cellular environment, releasing the peptide-free protein [27]. Using this approach, the folding of human superoxide dismutase (SOD1) during individual steps of the maturation process was studied [28,29]. Its misfolding is implicated in Lou Gehrig’s disease, leading to fatal motor neuron impairments. An alternate approach was provided by Ogino et al., who used pore-forming toxins (streptolysin O) to permeabilize the plasma membrane, followed by resealing the plasma membrane with Ca^2+^ to prevent cell death. This allows a sufficient amount of labeled protein to translocate into cultured human cells (Figure 1G). Thymosin β4 (Tβ4) was delivered via this method to 293F cells. The authors observed N-terminal acetylation of Tβ4, which occurred inside the cell as a posttranslational modification [30]. 

Electroporation is an efficient and recently employed method to transfer isotope-labeled protein into mammalian cells [5] The reversible permeabilization of the plasma membrane allows protein internalization via passive diffusion (Figure 1E). The Banci group expressed protein intracellularly within cultured human cells (Figure 1D) [31]. With this approach, it is possible to obtain atomic-resolution information pertaining to protein folding and maturation processes occurring immediately after protein synthesis in the cytoplasm, using NMR spectroscopy [32,33]. The expression and subsequent NMR analysis of proteins in insect cells was also reported (Figure 1C). For example, in-cell NMR spectra could be recorded using the sf9 cell/baculovirus system for four small model proteins (*Streptococcus* protein G B1 domain, *Thermus thermophilus* HB8 TTHA1718, rat calmodulin, and human HAH1 [34]). Electroporation was also successful in studying the phosphorylation pattern of the intrinsically disordered tau protein by in-cell NMR [35]. Here, disease-associated phosphorylation was immediately eliminated after delivery into human embryonic kidney (HEK)-293T cells. Further examples of in-cell NMR studies of protein phosphorylation are discussed in detail below.

More recently, interactions between unlabeled proteins and small molecules became accessible under in-cell NMR conditions. Here, for the first time, the interaction of unlabeled anti-apoptotic protein B-cell lymphoma 2 (Bcl-2) with the quercetin–alanine bioconjugate was studied in living human cancer cells utilizing saturation transfer difference (STD) and transfer NOESY (Tr-NOESY) NMR experiments [36].

## 2. In-Cell NMR and Small Molecules

In recent years, in-cell NMR was utilized for probing protein structures, protein folding, disulfide-bond formation, protein–protein and protein–small molecule interactions, and metal uptake in living cells [27,37,38,39,40]. In general, it is quite challenging to probe protein–protein and protein–small molecule interactions within living cells. Difficulties surrounding this include poor spectral quality caused by specific and non-specific interactions. However, recent developments showed the success of in-cell NMR in probing protein–small molecule interactions. 

### 2.1. Protein–Small Molecule Interactions

SMILI-NMR is an exciting example [15]. Once the target protein shows detectable and well-dispersed cross-peaks, NMR can be used to carry out target-engagement drug discovery inside the cell. A second example is the interaction of 12-kDa FK506-binding protein (FKBP12) within living cells with extracellularly administered immuno-suppressants [41]. Here, a cleavable CPP–ubiquitin–FKBP12 construct was prepared and the ^15^N-labeled fusion protein was expressed and purified from *Escherichia coli*. The labeled protein was transduced into HeLa cells. Following this, the CPP–ubiquitin was cleaved off by endogenous deubiquitinating enzymes of the HeLa cells. Subsequently, CPP aggregated, leaving^15^N FKBP12 as the sole soluble, labeled protein in the cytosol. Its cross-peak pattern was similar to ^15^N FKBP12 measured in vitro. This indicated that, in HeLa cells, the three-dimensional structure of FKBP12 was maintained and, therefore, the interaction with small molecules could be studied. Subsequently, in-cell NMR spectra were recorded after treatment with the immune-suppressants FK506 or rapamycin. Significant spectral changes were observed after administration of these drug molecules. Interestingly, the two spectra recorded in vitro and in cellulo had both similar features and subtle changes. Thus, some interactions may be correlated with the interaction observed only in living cultured cells [27,41].

In contrast to classical in-cell NMR experiments, in many cases, the protein cannot be labeled externally and then transduced into the cells of interest. Instead, two steps of production and isotopic labeling need to be carried out simultaneously. Special medium and labeling protocols are well established for bacteria and other organisms, including yeast and insect cells. The use of bacteria to overexpress protein within cells may lead to heavy background signals, caused by the concomitant labeling of other cellular components. To tackle this problem, the Dötsch group developed a scheme which reduced the background noise observed when studying the bacterial protein Nmer A. The activity of bacterial RNA polymerase can be inhibited by rifampicin but not by bacteriophage T7. Since protein expression was under the control of the T7 promoter, production of all endogenous bacterial proteins could be suppressed by rifampicin [6,7]. Nmer A plays a critical role in the bacterial pathway involved with mercury detoxification. The addition of Zn^2+^ to the NMR tube led to changes/disappearance of cross-peaks, thus highlighting the possible utility of this approach to investigate metal and drug binding. 

Recently, we showed the targeting of the bacterial chaperone “sensitive to lysis” (SlyD) to inhibit bacterial growth using a small molecule, with in-cell NMR spectroscopy [4]. Emergence of dangerous multi-drug-resistant strains of bacteria is one of the biggest threats to human health currently. With a continuous rise in antibacterial resistance, it was estimated that, by 2050, it may result in the death of 10 million people per year [42]. Among *Enterococcus faecium*, *Staphylococcus aureus*, *Klebsiella pneumoniae*, *Acinetobacter baumannii*, *Pseudomonas aeruginosa*, and *Enterobacter* species (ESKAPE) pathogens, Gram-negative bacteria are of particular concern, due to their increased ability to attain multi-drug resistance [43]. In the case of Gram-negative bacteria, many small molecules including antibiotics become ineffective, due to the presence of an outer polysaccharide layer and multi-drug efflux transporters. This led researchers to create a distinct class of novel antibacterial agents. For example, the heat shock proteins (HSPs) were targeted as potential molecules in cancer therapy [44,45]. Much attention was paid to target HSP90, leading to the identification of geldanamycin and radicicol; however, HSP60, HSP70, or other chaperones are less studied so far [46]. Therefore, we chose a small molecule, which was a metal-based coordination complex with a water-soluble organic moiety capable of crossing the cell-wall barrier and selectively targeting the bacterial chaperone SlyD.

**Figure 2:**
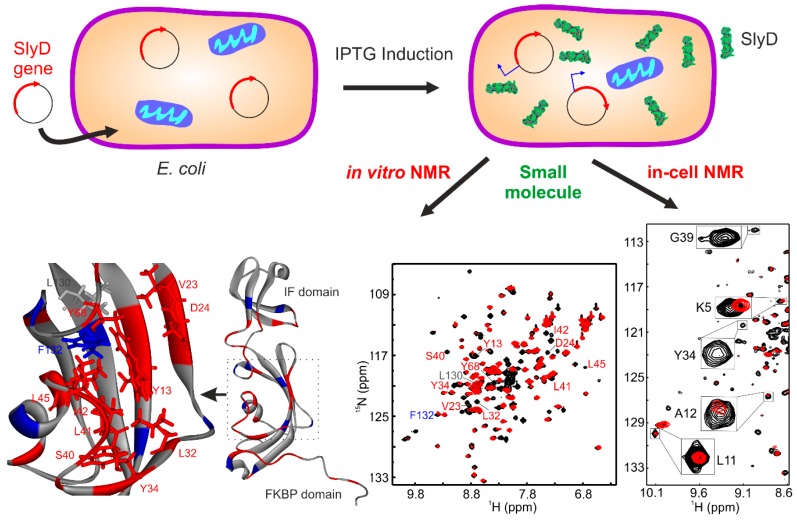
In-cell NMR study of protein–small molecule interactions. Here, a “sensitive to lysis” (SlyD)-containing plasmid was transformed into *Escherichia coli* and protein expression was initiated by isopropyl β-d-1-thiogalactopyranoside (IPTG) induction. These cells were further incubated with a small molecule (Cu^2+^ complex). The interaction of this small molecule with SlyD could be observed using in-cell NMR, and correlated with corresponding in vitro NMR studies, revealing the binding site in SlyD for this Cu^2+^ complex (adopted according to Reference [4]).

SlyD is a bacterial chaperone, and all prokaryotes and archaea express homologous proteins [47,48]. Thus, being unique to prokaryotes and archaea, SlyD represents a potential target against which to develop drug molecules. In these organisms, SlyD is involved in several biochemical pathways including the biosynthesis of [NiFe] hydrogenases, twin-arginine-mediated translocation (Tat transport), and metal storage/release. Additionally, SlyD exhibits both a peptidyl–prolylisomerase (PPIase) and chaperone activity, which prevents protein aggregation by binding to hydrophobic patches [47,48,49]. The PPIase and molecular chaperone activities [50] are located on two separate domains, whose cooperative interplay is required for full enzymatic activity [47,51,52,53,54]. The N-terminal tail contains the Ni^2+^ binding site followed by the FKBP binding domain. The FKBP domain harbors the active site of the PPIase, which is modulated by Ni^2+^, and the chaperone function is located on a domain that is inserted into the FKBP domain (Figure 2) [47,51,52,53,54].

Complete bacterial growth can be inhibited with a small molecule (anthracenyl terpyridine Cu^2+^ complex) at 2 µM [4]. In order to evaluate the role of SlyD in bacterial growth, we transformed *E. coli* with the plasmid containing the *SlyD* gene, which is under the control of T7 promoter. The expression of the *SlyD* gene can, thus, be initiated by isopropyl β-d-1-thiogalactopyranoside (IPTG). Overexpressed *SlyD* could restore bacterial growth, confirming that SlyD is involved in growth inhibition induced by the Cu^2+^ complex. This small molecule binds to SlyD with a dissociation constant (*K*_D_) ~50 µM with a 2:1 stoichiometry without inducing large conformational changes. In vitro NMR with ^15^N SlyD showed that this Cu^2+^ complex binds into the PPIase active site of the FKBP domain (Figure 2), as shown in earlier studies [54]. In-cell NMR spectroscopy confirmed with residue resolution that the Cu^2+^ complex also binds to SlyD inside bacteria, verifying that the complex can penetrate the cell wall and bind to SlyD when inhibiting cell growth. Interestingly, the small molecule also inhibited the growth of pathogenic bacteria from the category of ESKAPE pathogens. The half maximal inhibitory concentration (IC_50_) of the small molecule was below 1 µM for *Staphylococcus aureus* and *Pseudomonas aeruginosa* [4,55], showing the general applicability of the approach.

### 2.2. Small-Molecule Libraries

The Shekhtman group developed small-molecule interactor libraries NMR (SMILI-NMR). The interactions between two or more components of a biomolecular complex may be disrupted or enhanced by a few of these small molecules. The method provides atomic-level information and relies on the formation of a defined complex under in-cell NMR conditions. The approach was applied to the fujimycin (FK-506) binding protein (FKBP) and the FKBP rapamycin-binding domain of the mammalian target of rapamycin (FRB), a well-studied system for heterodimer formation, to screen those small molecules that can facilitate hetero-dimerization. In mammalian cells, one of the immune-modulatory systems (mitogenic responses) is constituted by rapamycin–FKBP–FRB interactions [56]. The labeled proteins were sequentially overexpressed using two compatible plasmids in *E. coli* under in-cell NMR conditions. To observe the NMR spectrum of FKBP or FRB, bi-complex (FKBP–FRB) formation was required. The X-ray structure of the FKBP–rapamycin–FRB ternary complex indicated the limited availability of the FKBP–FRB interaction surface. While the interaction between free FKBP and FRB is quite weak, with *K*_D_ values >50 μM, a well-defined ternary complex is formed when the FKBP–rapamycin complex binds to FRB (*K*_D_ ~12 nM). In their in-cell NMR experiments, either FKBP or FRB was labeled when the complex was formed inside the cells. This did not result in any visible spectrum. Addition of rapamycin to the cell suspension resulted in the appearance of FKBP resonances and, for 32 out of the 107 residues, changes with respect to free FKBP could be detected. Many resonances in FRB residues also changed upon performing the reverse experiment [15]. Ascomycin is a competitive inhibitor of rapamycin. It binds to FKBP with 1.4 nM affinity and has no known affinity with FRB. However, the ascomycin–FKBP complex binds to FRB with lower affinity [57]. Addition of ascomycin to the aforementioned dual-plasmid system resulted in the appearance of the FKBP/FRB NMR spectrum. In the next step, the authors screened a library of small molecules of 289 dipeptides with SMILI-NMR. The peptides were selected as drug candidates from the literature, based on their facile and cost-effective preparation, as well as their ability to be imported into prokaryotic and eukaryotic organisms through naturally occurring transport systems. Screening of a matrix of 17 × 17 peptides resulted in the identification of various combinations that showed completely different behavior when compared to the rapamycin-induced ternary complex. Addition of these dipeptides resulted in extreme line broadening and the disappearance of some peaks in the NMR spectrum. Later on, the addition of low concentrations of Ala–Glu resulted in the same interactions with FKBP, suggesting that FKBP and FRB hetero-oligomerization can be facilitated by this peptide. Thus, SMILI-NMR could screen the protein–small molecule interactions within the cellular environment using high-resolution NMR as a readout [15]. 

## 3. In-Cell NMR Observation of Metabolic Processes Using Hyperpolarization

NMR spectroscopy provides considerable opportunities for collecting diverse and unique information on cellular processes due to its non-invasive nature [58], rendering it highly suitable for studying the fate of metabolites and biochemical pathways in situ. In order to raise the detection limit of this inherently insensitive technique, hyperpolarization NMR methods—mainly dissolution dynamic nuclear polarization (d-DNP) and *para*-hydrogen-induced polarization (PHIP)—were devised as tracer techniques to follow the fate of detectable, small molecular probes for the visualization of cellular functions that are not easily observable by other means. Furthermore, NMR signals from these molecules can be enhanced by several orders of magnitude via the combined use of hyperpolarization (HP) and isotope enrichment. The NMR signal enhancements achieved using these methods are often sufficiently high to track endogenous molecules at physiological concentrations. Among all the potentially suitable target molecules for these specialized experiments, hyperpolarized pyruvate, a metabolite whose cell biochemistry lies at the interface between catabolic and anabolic metabolism, is the most widely studied probe. This is mainly due to its (a) high hyperpolarizability, (b) rapid cellular uptake, and (c) central biochemical position as a key intermediate in several biochemical pathways. In addition, a host of other probes emerged in the meantime that can also be used to characterize the phenotype of cells under a particular set of conditions (Table 1).

Experimentally, the method, which can lead to a nuclear sensitivity enhancement of up to five orders of magnitude, works as follows: a frozen solution (T ~1.1 to 1.5 K) of the sample to be analyzed is polarized in the presence of a radical molecule, e.g., trityl (triphenylmethyl, “Trityl OX063”) or the nitroxide-based radical 2,2,6,6-tetramethylpiperidin-1-yl)oxyl (TEMPO) [59], with microwave (MW) irradiation (spin polarization is transferred via the DNP mechanism from electrons to nuclei upon microwave irradiation at or near the Larmor frequency of the radical electron [60,61]) using a specifically designed DNP polarizer. Subsequently, the sample is thawed, dissolved in a suitable hot solvent, and then transferred to a conventional liquid-state NMR spectrometer for detection [62]. In solids, DNP is known to occur via a number of different mechanisms known as the solid effect, thermal mixing, and the cross effect [60]. Depending on the experimental conditions used in each d-DNP experiment, e.g., radical type, substrate, and solvent (among others), the contribution of each of these effects to the observed polarization enhancement differs.

The longitudinal relaxation time (*T*_1_) of the hyperpolarized nuclei is crucial in dissolution DNP. To preserve the nuclear polarization acquired in the solid state, polarized samples need to be thawed and transferred to the NMR spectrometer faster than nuclear *T*_1_ spin–lattice relaxation. Typical dissolution DNP samples experience a so-called “transfer time” as they are moved from the polarizing magnet to the NMR spectrometer. During this period, the sample is often exposed to low magnetic fields, which are typically on the order of ca. 0.5 mT. Given that nuclear *T*_1_ times are generally shorter at low magnetic fields—and even more so in the presence of radicals—a fast transfer of the sample, as well as the elimination of stable free radicals in solution, is crucial to reduce polarization losses [63]. The use of a “magnetic tunnel” for transfer and/or the addition of a radical scavenging agent, e.g., ascorbate (vitamin C), to the sample during the dissolution step were shown to alleviate polarization losses during and immediately after the transfer. Attempts to shorten the transfer time include construction of a dedicated “hybrid” spectrometer, comprising a single dual-isocenter superconducting magnet featuring regions of different magnetic field strengths suitable for both polarization and NMR detection [64] or, alternatively, a “shuttle” DNP spectrometer comprising a two-center magnet [65].

A significant number of d-DNP-based investigations were carried out in recent years to probe the metabolic behavior of tissues, e.g., heart, liver, and tumor cells, and to study the fate of individual metabolites both in vitro and in vivo [66]. In addition, metabolite molecules hyperpolarized using the d-DNP method were used to probe a variety of different biochemical pathways. For example, the enzymatic conversion of pyruvate to lactate, acetylcarnitine, citrate, and glutamate was tracked in real time employing [2-^13^C] pyruvate, in isolated perfused heart tissue, to study healthy and pathological states [67]. Hyperpolarized probes were also used to track intracellular pathways of short-chain fatty acids and ketone body metabolism in real time. A butyrate probe visualized the flux of fatty acids to acetoacetate and several tricarboxylic-acid-cycle intermediates in cardiac muscle cells [68]. In addition, hyperpolarized [1-^13^C] pyruvate was used as a clinical diagnostic tool in metabolic imaging to characterize differences between healthy tissue and tumor cells [69]. Further applications include the study of enzyme kinetics [70], biosynthetic pathways [70], and the detection of lowly populated reaction intermediates [71]. More recently, the *para*-hydrogen-induced hyperpolarization method was also introduced for the signal amplification of metabolites and the subsequent tracking of their biochemical pathways in both healthy and pathological forms of tissue. Up to this point, however, only a few very recent examples exist in the literature where the method was successfully employed to hyperpolarize metabolites for in-cell NMR studies [72,73,74].

In the following subsections, we give an overview of hyperpolarization NMR methods—in particular, dissolution dynamic nuclear polarization (d-DNP) and *para*-hydrogen-induced nuclear polarization (PHIP)—and a few selected examples of hyperpolarization-assisted in-cell NMR observations of metabolic processes are highlighted in a conceptual manner. The section concludes with an outlook into future perspectives of this emerging, yet still relatively novel, area of NMR research.

### 3.1. Dissolution DNP Application to Metabolic Pathways and Biological Functionality

The number of applications of heteronuclear d-DNP for the study of living cellular systems is vast [66]. For example, the real-time tracking of metabolic conversion using hyperpolarized NMR is particularly suitable for the observation of metabolic reaction networks, provided that conversion rates are high and that the obtained levels of hyperpolarization are significant. In this context, glycolysis was identified as an adequate metabolic process to be studied with d-DNP, given its overwhelming biochemical importance and its central role in a variety of different biochemical reaction routes. For example, enzymatic reaction mechanisms, bottlenecks, and off-pathway reactions were probed using hyperpolarized carbohydrates, i.e., [2-^13^C] fructose and [U-^13^C, U-^2^H] glucose, as substrates [66]. Chemical detail in the observation of pathway reactions extends to the distinction of isomers and their susceptibility to enzymatic turnover. The use of site-specifically labeled [2-^13^C] fructose, for example, permitted the real-time observation of probe flux during gluconeogenesis, as well as the formation of non-productive off-pathway intermediates, such as dihydroxy acetone phosphate hydrate [75]. A recent approach combined hyperpolarized dynamic measurements with metabolite extraction, isotopomer evaluation, and flow analysis [76]. This approach measured pyruvate metabolism in living cells to obtain quantitative data of several biochemical pyruvate pathways in different cell types.

The existence of different interlocked pathways, all featuring a similar set of key metabolites, makes it difficult to predict how cellular physiology and intracellular metabolism respond to the modification of individual genes [77]. The use of hyperpolarized NMR spectroscopy to study genetically well-defined and homogeneous cell suspensions shows promise in studying the cellular response to genetic modifications. For example, the two *E. coli* strains, BL21 and K-12, show strong differences in the reaction progression of their pentose phosphate pathways. In the BL21 strain, a reactive intermediate accumulates, and is responsible for covalent modifications observed for the recombinant proteins expressed within this strain [78]. Genome alignment techniques prove that the gene encoding for lactonase—the enzyme which catalyzes the hydrolysis of 6-phosphogluconolactone—is absent in the BL21 strain due to a deletion. Such molecular phenotypes can be observed in the absence of phenotypic variations [79]. Metabolic differences in different cell types were recently compared in human cells by tracking the glycolytic pathway [80]. Ratiometric measurements of lactate and pyruvate signals in two different proliferating cell types were used to non-invasively detect differences in the cytosolic redox state. In the cytosol, lactate and pyruvate form a redox pair, whose equilibration rate depends crucially on the ratio of oxidized to reduced nicotinamide adenine dinucleotide (NAD^+^/NADH) in the cytosol. PC3, a specific prostate cancer cell line, showed a fourfold increase in the intracellular ratio of free cytosolic NAD^+^/NADH in comparison with breast cancer cells in an experiment that used hyperpolarized glucose as a reporter metabolite. The increase in the ratio of NAD^+^ versus NADH reflects a distinct metabolic phenotype consistent with previously reported alterations in the energy metabolism of prostate cells. In a relatively recent study, the importance of hyperpolarized NMR probes as tools for functional studies involving the human genome was underlined by observing human cell types differing only in the mutational status of the enzyme isocitrate dehydrogenase 1 (IDH1), using hyperpolarized [1-^13^C] alpha-ketoglutarate as a molecular reporter. IDH1 catalyzes the decarboxylation of cytosolic isocitrate to α-ketoglutarate. Specific mutations in IDH1 result in its ability to catalyze the NAD phosphate (NADPH)-dependent reduction of α-ketoglutarate to (*R*)-2-hydroxyglutarate, an onco-metabolite [81]. As a consequence, isogenic glioblastoma cells, differing only in the status of IDH1, show differences in the conversion of the hyperpolarized α-ketoglutarate to (*R*)-2-hydroxyglutarate, as probed by changing hyperpolarized NMR signal intensities.

### 3.2. Following Metabolism in Living Microorganisms Using Hyperpolarized ^1^H NMR

As mentioned before, most d-DNP-based in-cell NMR studies focus on hyperpolarizing nuclei with low gyromagnetic ratios (γ), given their relatively long spin–lattice (*T*_1_) relaxation times, e.g., non-protonated ^13^C or ^15^N nuclei in small molecules exhibiting short rotational correlation times (*τ*_c_). Nevertheless, advantages can also result from observations based on hyperpolarizing and observing high-γ nuclei, e.g., protons. For example, ^1^H signal intensities should be, on average, approximately 16-fold higher as compared with ^13^C, given the fourfold higher gyromagnetic ratio of protons. While this gain is moderated by a concomitant increase in spectral noise, the indisputable fact that state-of-the-art ^1^H-observation hardware is widely available and represents the most mature across all in vivo NMR technologies, might make these observations more worthwhile. Lastly, instances may arise where ^1^H-based detection provides a better chemical discrimination than ^13^C-based methods. In fact, spontaneous enhancements in the ^1^H-NMR spectra of hydrogen nuclei covalently bound to hyperpolarized ^13^C nuclei were reported a while ago [82,83]. Heteronuclear cross-relaxation effects arising in rapidly tumbling small molecules were identified as the mechanism responsible for this spontaneous polarization transfer. Given that, in such experiments, hyperpolarization can be stored in a relatively slowly relaxing nucleus that shares its hyperpolarization with a neighboring proton, opportunities arise from using these latter signals to monitor enzymatic turnover.

Using ^1^H NMR detection, two such processes were recently studied by Frydman and co-workers to yield successful results [84] using both solutions of purified enzymes in vitro and suspensions of intact cells. The substrate in each of these studies was hyperpolarized [^13^C] pyruvate, and the enzymatic processes targeted were (a) the production of acetaldehyde following the addition of hyperpolarized [U-^2^H_3_,2-^13^C] pyruvate either to samples containing pyruvate decarboxylase (PDC) purified from *Saccharomyces cerevisiae* or, alternatively, to cultures of *S. cerevisiae* fermenting glucose, and (b) the generation of formic acid due to the activity of pyruvate formatelyase (PFL), measured in cultures of anaerobic *E. coli* following the addition of hyperpolarized [1-^13^C] pyruvate. In these enzymatic reactions, the formation of new covalent bonds between the hyperpolarized ^13^C nucleus and protons in the reaction products, i.e., acetaldehyde and formate, allowed the authors to transfer hyperpolarization using either insensitive nuclei enhanced by polarization transfer (INEPT)-type pulse sequences or by spontaneous cross-relaxation. Features that favored the execution of such reversed INEPT experiments included (i) the possibility to transfer magnetization between heteronuclei separated by multiple bonds (something that cross-relaxation is very inefficient at doing); (ii) the possibility of incorporating coherence-selection pulsed field gradients (PFG) to efficiently eliminate background ^1^H signals of endogenous metabolite molecules; and (iii) the possibility of acquiring ^1^H NMR spectra exhibiting a signal-to-noise ratio (SNR) which is approximately an order of magnitude higher than the SNR obtained using solely spontaneous magnetization transfer by cross-relaxation. In particular, using ^1^H-detected INEPT spectroscopy allowed the detection of the acetaldehyde produced from hyperpolarized pyruvate. The authors also observed, however, that the strong signal enhancement achieved by the reversed INEPT experiment came, in part, at the expense of depleting all of the ^13^C polarization in a single acquisition. It was, hence, deduced that lower levels of polarization transfer can be delivered using the INEPT sequence at the expense of not being able to employ more complex procedures, which would otherwise “waste” hyperpolarization generated via the DNP mechanism. It was suggested that a possible route to preserve the bulk ^13^C hyperpolarization while using INEPT would be to use sequences that selectively excite the carbon nuclei of the product while avoiding excitation of the reactant ^13^C coherences.

Subsequently, Frydman and co-workers explored the same biochemical process in cultures of *S. cerevisiae* using proton detection. Although these microorganisms can be grown on pyruvate as the sole source of carbon, pyruvate does not permeate the plasma membrane during glucose fermentation. Undissociated pyruvic acid, however, rapidly crosses the plasma membrane of glucose-fermenting *S. cerevisiae*. In this context, a recent d-DNP NMR study using carbon detection demonstrated that rapid diffusion of undissociated HP [1-^13^C] acetic acid into glucose-fermenting *S. cerevisiae* occurred, in particular, at low extracellular pH [85]. After entering the cell, pyruvic acid is rapidly decarboxylated by cytosolic PDC, a process which is pronounced during the exponential stages of cell growth. The product, acetaldehyde, is present in a low equilibrium concentration and rapidly reduced to ethanol. The accumulation of acetaldehyde in *S. cerevisiae* cultures was observed with acidification of the cytosol, which was attributed to the inhibition of the enzyme alcohol dehydrogenase combined with a shift in cytosolic pH to values closer to PDC’s pH optimum of 6.0 [86]. The ^1^H NMR results obtained by Frydman and co-workers were able to corroborate this feature via detection of a relatively weak acetaldehyde ^1^H signal found in NMR spectra of mid-exponential yeast cultures following incubation in acetate buffer at pH 4.5 and prior exposure to hyperpolarized pyruvic acid.

In a final step, the wider applicability of spontaneous polarization transfer for investigating cell metabolism was demonstrated in a study investigating the activity of PFL in *E. coli* cells. PFL rapidly metabolizes pyruvate in anaerobic *E. coli* cultures, a process that is particularly heightened when pyruvate is the main carbon source. Consistent with results obtained from direct ^13^C detection, Frydman and co-workers observed the rapid uptake and breakdown of pyruvate as a pronounced formate ^1^H signal. In contrast to the relatively weak acetaldehyde signal detected in *S. cerevisiae* cultures after the addition of HP pyruvic acid, the build-up and decay of the formate signal was observed on a single, scan-by-scan basis. The authors explained this higher signal-to-noise ratio by the rapid uptake of pyruvate and the accumulation of formate as a metabolic end product, characteristic of anaerobic *E. coli* cells.

In summary, Frydman and co-workers were able to demonstrate that ^13^C-based dissolution DNP, combined with both spontaneous and INEPT-driven polarization transfer from carbon to protons, provides a clear ^1^H signature of the enzymatic processes studied. They eventually concluded that this feature can help decipher NMR-encoded metabolic information in spectra acquired *in cellulo*. Even though metabolic fluxes from pyruvate to acetaldehyde in *S. cerevisiae* [85] and of pyruvate to formate in *E. coli* [79] were measured in the past using d-DNP-enhanced ^13^C NMR in vitro, close signal proximity and subsequent signal overlap between the metabolic products and their direct precursors complicated these measurements in vivo to a significant extent. Also, the spontaneous transfer to ^1^H turned out to be particularly superior to other methods, as it allowed the detection of hyperpolarized ^1^H signals without the need for chemical manipulation of the probe molecule prior to the experiment, or modifications of the dissolution method.

## 4. *para*-Hydrogen-Induced Hyperpolarization Side-Arm Hydrogenation (PHIP-SAH) Method for the Detection of Cell Metabolism

As mentioned before, *para*-hydrogen-induced polarization (PHIP) is a chemistry-based hyperpolarization technique which, to a certain extent, is easier to handle and more straightforward to use when compared with DNP. This is due to the fact that (i) no additional NMR set-up extension, i.e., polarizer equipment, is needed, and (ii) polarization times are significantly shorter. The main drawback of the method in the context of in-cell NMR studies, however, is the limited availability of unsaturated precursor molecules with respect to the desired target compounds to be studied; for instance, nuclear spin-polarized acetate and pyruvate cannot be obtained by direct incorporation of the *para*-hydrogen molecule. The advent of non-hydrogenative PHIP (NH-PHIP; see below) only partially resolved this problem, given that the obtained levels of hyperpolarization in the target compounds are significantly lower as compared to using, for example, the adiabatic longitudinal transport after dissociation engenders net alignment (ALTADENA)-PHIP method. Furthermore, not all substrates of interest are suitable for NH-PHIP, given that certain requirements with respect to their molecular and electronic structure must also be fulfilled in this case.

Molecular dihydrogen occurs in the form of two nuclear spin isomers, i.e., the *ortho*- and the *para*-isomer, featuring either symmetric (triplet) or antisymmetric (singlet) nuclear spin states, respectively. Due to the three-fold degeneracy of the triplet state, *ortho*- and *para*-isomers are populated in a ratio of 3:1 under ambient conditions [87]. Interconversion between the two is symmetry forbidden and occurs, hence, at a negligibly small rate. However, when H_2_ gas is flowed through an appropriate apparatus comprising a paramagnetic catalyst, i.e., activated charcoal, at low temperatures, a high enrichment of the *para* spin isomer can be achieved and observed at standard temperatures [88] due to this form being lower in energy.

When applying the *para*-hydrogen-induced polarization (PHIP) method, nuclear spin hyperpolarization is accomplished by transferring the high spin order of the *para*-hydrogen molecule to the substrate of interest. Typically, PHIP applications involve the direct incorporation of *p*-H_2_ into unsaturated organic molecules, i.e., molecules containing either carbon–carbon double or triple bonds, in the presence of an appropriate hydrogenation catalyst. The new chemical environment experienced by the two protons upon hydrogenation breaks the singlet symmetry and renders the spin system detectable by NMR. Depending on whether the reaction is carried out at high or low magnetic field, the methods are named *para*-hydrogen and synthesis allow dramatically enhanced nuclear alignment (PASADENA) or ALTADENA, respectively [89,90]. In PASADENA experiments, the *para*-hydrogen symmetry is broken upon hydrogenation due to the distinct chemical shift environment of the two incorporated protons at high magnetic field. Thus, the NMR spectrum shows two antiphase multiplets. In ALTADENA experiments, the singlet state becomes selectively polarized, given that the chemical shifts are essentially the same when *para*-hydrogen is incorporated into the substrate at low magnetic field. In this case, the NMR spectrum is characterized by two hyperpolarized in-phase resonances of opposite sign. In the case that other magnetically active nuclei are present in the substrate, i.e., ^13^C, ^15^N, or ^19^F, scalar and/or dipolar coupling interactions can cause the transfer of the initially induced proton hyperpolarization to other regions of the substrate molecule, in particular when the hydrogenation step is carried out at low field (ALTADENA); this phenomenon was also observed using other hyperpolarization methods [91,92].

Despite the relatively high degree of both homo- and heteronuclear polarization that can be achieved with PHIP, the method is not generally applicable to all molecules, i.e., metabolites, unless characteristic hydrogenation precursor modifications are applied (see below). More recently, a related methodology known as signal amplification by reversible exchange (SABRE) or NH-PHIP [93] was developed to polarize substrates without having to perform the final hydrogenation step, i.e., the physical transfer of the two protons of the dihydrogen molecule to the substrate. During a SABRE experiment, substrate and *para*-hydrogen nuclei experience transient contact interactions via the metal center of a labile catalyst–dihydrogen complex, e.g., [Ir(H)_2_(PCy_3_)(substrate)_3_][BF_4_], which is formed via the reaction of [Ir(COD)(PCy_3_)(MeCN)][BF_4_]—where “Cy” is cyclohexyl and “COD” is cyclooctadiene) with *para*-H_2_ and an excess of the substrate to be polarized. While the complex is formed, nuclear polarization is transferred from the *para*-hydrogen-derived protons to the molecule of interest. After the polarization transfer step, the chemically unmodified polarized substrate is released. So far, pyridine is the most widely studied SABRE substrate, and more than 10% of proton polarization was achieved in this case [94]. The method is strikingly similar to the ALTADENA experiment described above in that the polarization of the substrate occurs at low magnetic field. The magnetic field dependence of SABRE-derived signal enhancements appears to change very little with substrate type or position of the protons, suggesting that the extent of polarization depends primarily on the scalar coupling between two *para*-hydrogen-derived protons and not on the scalar coupling between *para*-hydrogen-derived and substrate protons within the hydrogenation complex [95,96].

Recently, Aime and co-workers presented a new addition to the *para*-hydrogen method, which allows the generation of high levels of nuclear polarization using substrates normally inaccessible to the *para*-hydrogen method, which can subsequently be used for hyperpolarization-assisted NMR studies in cellulo [74]. In particular, their results demonstrate that PHIP can be induced in nuclei of molecules such as acetate and pyruvate—and, in principle, other carboxylic acids as well—using precursors containing an unsaturated side-arm moiety capable of hydrogenation that can be hydrolyzed to yield the hyperpolarized target products. The reported method, named PHIP-SAH, relies on the following steps: (i) functionalization of the target acidic molecule with an unsaturated alcoholic group (i.e., vinyl or propargyl alcohol; using propargyl alcohol as a removable synthon to generate PHIP on ^13^C resonances markedly widens the applicability of this approach); (ii) *para*-hydrogenation of the unsaturated ester; (iii) heteronuclear polarization transfer from the former pair of *para*-hydrogen protons to the carboxylate ^13^C signal by applying magnetic field cycling, where the magnetic field is cycled between the Earth’s magnetic field (hydrogenation step) and nearly zero-field (polarization transfer step) using concentric cylinders made of µ-metal, thereby increasing the extent of polarization transfer to carbon nuclei; (iv) release of the alcohol moiety via hydrolysis to obtain the polarized ^13^C-carboxylate-containing product.

**Figure 3:**
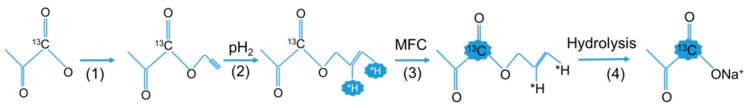
Diagram of the *para*-hydrogen-induced hyperpolarization side-arm hydrogenation (PHIP-SAH) procedure. (1) Functionalization of the carboxylate group with the side-arm; (2) *para*-hydrogenation of the unsaturated alcohol; (3) transfer of *para*-hydrogen spin order to the ^13^C spin of the carboxylate group; (4) cleavage of the side-arm. The yellow background indicates reaction steps taking place in the organic phase, while the blue background indicates that the molecule is dissolved in the aqueous phase. This figure was adopted according to Reference [98].

In summary, these findings open up a very interesting perspective for the use of *para*-hydrogen-based procedures for the generation of hyperpolarized, biologically relevant molecules, as demonstrated by recent NMR studies carried out in living cells using PHIP-SAH. Examples include the real-time detection of the response of the heart to altered metabolism [73], as well as the study of tumor cell metabolism using hyperpolarized ^13^C-labeled pyruvate [72], in addition to the observation of the metabolic transformations of hyperpolarized lactate in vitro [72]. The fact that means to further enhance the efficiency of hyperpolarizing small organic molecules via PHIP-SAH were identified recently (Figure 3) will most likely lead to an even wider applicability of this very promising hyperpolarization method [97].

## 5. In-Cell NMR and Posttranslational Phosphorylation

The most common posttranslational modifications of proteins by their respective kinases is the phosphorylation of serine, threonine, and tyrosine, as well as histidine and aspartate residues, in bacteria and fungi [99]. The addition and removal of the phosphate group by phosphatases plays a key role in the regulation of biological processes. Phosphorylation patterns modify the structure and stability of proteins, in addition to their localization and specific interactions with binding partners. Anomalous phosphorylation events are the basis for many human diseases. Thus, deciphering the phospho-code remains a major research effort.

Mass spectrometry (MS) is one of the techniques of choice to identify posttranslational modifications of the primary protein sequence due to its extreme sensitivity and high resolution. MS relies on enzymatic digestion of the protein of interest (usually with trypsin), followed by subsequent peptide analysis. However, multiple phosphorylation events which are in close proximity are difficult to analyze in terms of their exact location. MS/MS methods can solve this problem. The labile nature of the phosphate groups, however, might further hamper the analysis. Thus, mass spectrometry analyses benefit from further biochemical verifications. NMR spectroscopy is the method of choice to correlate the phosphorylation patterns with conformational changes of the protein, and in-cell NMR extends these investigations to the natural environment of the protein of interest.

### 5.1. In-Cell NMR within *Xenopus laevis* Oocytes

Selenko and co-workers utilized time-resolved NMR to monitor the sequential phosphorylation of Ser111 and Ser112 of the HIV SV40 large T antigen regulatory region [26], catalyzed by casein kinase 2 (CK2) [100]. The nuclear-import properties are modulated by these events, primarily mediated by the proximal monopartite nuclear localization sequence. The complete regulatory sequence was incorporated into the model CK2 substrate (XT 111–132 GB1). Here, the GB1 domain (B1 domain of streptococcal protein G) acts as a solubility enhancer. In the in vitro kinase assay monitored by NMR spectroscopy, CK2 firstly phosphorylated Ser112 to the maximum extent, followed by the initiation of Ser111 phosphorylation. In the S112A variant, the kinase reaction rate for the phosphorylation of S111 by CK2 was greatly reduced. Thus, phosphorylation of Ser111 depends on the pre-modification of Ser112 and the presence of a negative charge at this position. Conversely, substitution of Ser112 with aspartate revealed rapid phosphorylation of Ser111, whereas alanine at position 111 had no effect on Ser112 phosphorylation. The authors observed the same sequential phosphorylation when XT 111–132 GB1 was mixed with *Xenopus laevis* egg extract and monitored by NMR spectroscopy [20]. Finally, ^15^N-labeled XT111–132 GB1 was microinjected into freshly prepared *Xenopus laevis* oocytes, then subsequent time-resolved in-cell NMR experiments confirmed the sequential phosphorylation of Ser112 followed by Ser111 in cellulo [26].

A second time-resolved in-cell NMR approach within *X. laevis* oocytes was reported following multiple phosphorylation of the “unique domain” of non-receptor tyrosine kinase c-Src [101]. An ^15^N-labeled fragment of 85 residues containing the intrinsically disordered part of human c-Src was injected into the oocytes yielding an almost identical NMR spectrum compared with the in vitro spectrum with one exception: Ser17 was phosphorylated in both intact oocyte extracts. The authors then performed the time-resolved NMR experiment with extracts obtained from unfertilized *X. laevis* eggs. In this experiment, various peaks appeared/disappeared at different time points, revealing the sequential phosphorylation of three Ser residues (S17, S75, and S69). The cross-talk between the respective kinases and phosphatases was further evaluated using specific inhibitors in the oocyte extracts [101].

### 5.2. NMR Studies with Cell Extracts

Another NMR approach to get as close as possible to the cellular environment is to study isotope-labeled proteins in cell extracts, for example, from *Xenopus laevis* oocytes [101,102], HeLa [24,103], U2OS [24], HEK-293 [24], *Drosophila melanogaster* embryos [24], parathyroid glands [104], A2780 [103], RCSN-3 [103], B65 [103], or SK-N-SH [103]. A careful preparation preserves the proteome of the respective cells, which might even include the differentiation or cell-cycle state.

Using time-resolved NMR spectroscopy in vitro and with cell lysate, multisite phosphorylation on E26 transformation-specific (ETS)-like gene 1 (Elk-1) and corresponding extracellular signal-regulated kinase (ERK) activation was investigated [102,105]. In general, multisite phosphorylation can regulate various transcription factors involved, for example, in nuclear import and export, protein turnover, gene activation, and various protein–protein interaction (see references in Reference [105]). However, the functional role of individual phosphorylation events and their dynamics remains poorly understood. Elk-1, SAP-1, and Net form a ternary complex factor (TCF) subfamily of ETS-domain transcription factors. The kinetics of multisite phosphorylation of the Elk-1 transactivation domain (TAD) was recorded with NMR spectroscopy using recombinant ERK2 [102,106]. These time-resolved experiments revealed that phosphorylation of Thr369 and Ser384 occurred faster than the modification of Thr354, Thr364, and Ser390, whereas slower modifications were observed for residues Thr418, Ser423, and Thr337. Based upon the Michaelis–Menten constants, the authors proposed a model where an increase in the rate of phosphorylation of the “slow site” occurs after removal of fast and intermediate phosphorylation sites, which they experimentally confirmed by site-directed mutagenesis. This allowed the authors to refute certain hypotheses, for example, that fast-site phosphorylation primes later modification events [102].

**Figure 4:**
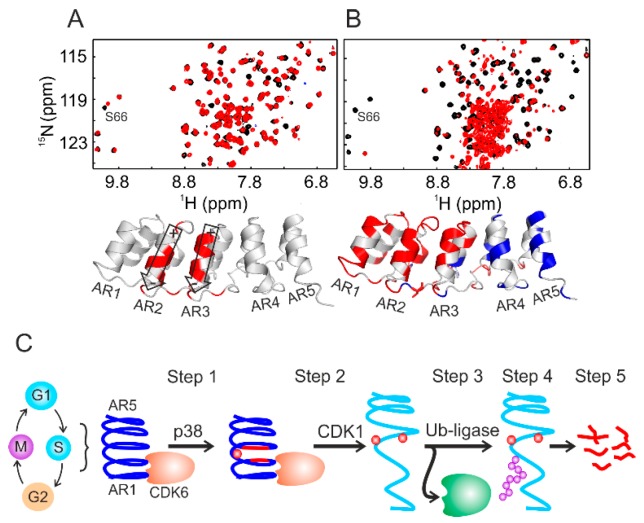
Following cell lysate-induced protein phosphorylation using NMR spectroscopy. (**A**) Superposition of two-dimensional (2D) ^1^H–^15^N HSQC spectra of phosphorylated p19^INK4d^ at Ser66 (red) and non-phosphorylated protein (black). (**B**) Superposition of 2D ^1^H–^15^N HSQC spectra of doubly phosphorylated p19^INK4d^ at Ser66 and Ser76 (red) and the non-phosphorylated form (black). The bottom panels in (**A**) and (**B**) represent the backbone NMR chemical-shift mapping on the p19^INK4d^ structure. (**C**) Summary of the fate of p19^INK4d^ during the cell cycle controlled by phosphorylation and ubiquitination. This figure was adapted from Reference [24].

Using the combination of NMR spectroscopy and cell biology, we delineated the periodic oscillation of p19^INK4d^ through the human cell cycle [24]; p19^INK4d^ undergoes a two-step phosphorylation, strictly coupled to the gap 1/synthesis (G1/S) cell phase transition. In mammalian cells, this transition is primarily regulated by a transcription factor of the E2 factor (E2F) family (E2F1 to E2F8). Most E2 factors form an inactive dimer complex with the distantly related dimerization partner (DP) protein. These E2F–DP complexes stall the cells in the G1 phase. Upon entry into S phase, the cyclin-dependent kinase complex CDK4/6–cyclin D gets hyper-phosphorylated and disrupts E2F–DP complexes. The thereby activated E2Fs are necessary for the gene expression required for the G1/S transition [107]. The kinase activity of CDK4/6 itself is inhibited by members of the CIP/KIP and INK4 protein families. The here reviewed p19^INK4d^ belongs to the latter class of inhibitors also comprising p16^INK4a^, p15^INK4b^, and p18^INK4c^ [108,109]. P19^INK4d^ consists of five ankyrin repeats (AR), and AR1 and AR2 bind to CDK4/6 to facilitate inhibition. Interestingly, the regulatory sites for phosphorylation (Ser66/Ser76) and ubiquitination (Lys62) are located opposite to the CDK binding interface. In order to follow phosphorylation, ^15^N-labeled p19^INK4d^ was incubated with lysates prepared from the exponentially growing HeLa, U2OS, HEK-293 cells or from *Drosophila melanogaster* embryos. Two-dimensional (2D) ^1^H–^15^N HSQC-based NMR analyses indicated the pronounced chemical-shift change of the Ser66 resonance upon phosphorylation (Figure 4A). Most of the NMR resonances remained at their native positions, indicating that p19^INK4d^ largely retained its folded conformation. Ser76 did not get phosphorylated by these lysates.

The periodic oscillation of p19^INK4d^ during the cell cycle has its maximum during the S phase. Therefore, ^15^N-labeled p19^INK4d^ was also incubated with S-phase-synchronized HeLa cell lysate. Surprisingly, the NMR cross-peaks, from residues belonging to AR1, AR2, and AR3, disappeared from their native position and clustered around 8 ppm along the proton dimension (Figure 4B), resembling an unfolded protein conformation. AR4 and AR5 remained folded. These structural rearrangements were induced by phosphorylation of Ser76, which turned out to be a sequential process after Ser66 phosphorylation and strictly cell-cycle-dependent. Adding specific inhibitors to the lysates before the NMR analyses identified p38 and CDK1 as kinases for Ser66 and Ser76, respectively. Further investigations, including cell-lysate NMR, revealed that phosphorylation of Ser66/Ser76 caused dissociation of the p19^INK4d^/CDK6 complex, as well as exposure of Lys62 for subsequent ubiquitination and degradation, as proposed earlier from cell biology studies [110,111]. The irreversibility of the latter step (Figure 4C) ensures, by proxy, the directionality of the cell cycle [24].

Using similar cell lysis NMR approaches [104], we disentangled the 1984 paradigm of parathyroid hormone (PTH) phosphorylation. PTH is an 84-residue peptide which controls blood calcium homeostasis. In 1984, phosphorylation of PTH was proposed based upon its high performance liquid chromatography (HPLC) elution profile being different to that of native PTH [112]. Later on, various modified forms of PTH were identified within human blood. The concentration of the modified form may rise up to 65% in patients suffering from cancer of the parathyroid glands [113,114,115,116]. In order to remain close to biological conditions, we utilized the cell lysate from bovine parathyroid gland and HEK-293 cells to incubate ^15^N PTH. The convergent analysis of subsequent one-dimensional (1D) ^31^P and 2D ^1^H–^15^N NMR, autoradiography, and matrix assisted laser desorption ionization-time of flight (MALDI-TOF) data revealed that, out of the seven serine residues of PTH, Ser1, Ser2, and Ser 17 got selectively phosphorylated. This posttranslational modification prevented in cellulo activation of PTH receptors 1 and 2 [104], which are G-protein-coupled receptors and drug targets for osteoporosis treatment [115]. PTH consists of three distinct functional regions: residues 1–17 are responsible for activation of the PTH receptors, residues ~18–34 facilitate binding to the extracellular domain (ECD) of the class B G protein coupled receptor [117,118,119], and the role of the intrinsically disordered residues ~34–84 might relate to the formation of functional PTH amyloids as a storage form of the hormone in secretory granules, which is currently under debate [120,121]. Our finding that phosphorylated PTH can still bind to the ECD of the PTH receptor without activation is in line with the recently reported crystal structure of the PTH1 receptor in complex with a PTH variant [115], where the N-terminus of PTH binds deeply into the trans-membrane domain of the receptor, probably resulting in the distortion of the domain by the three negative charges of phosphorylated serines 1,2, and 17.

## 6. Divide and Conquer by In-Cell and Cell-Lysate NMR

With an increasing complexity of proteins and protein assemblies, high-resolution structural biology methods are getting closer to their limits. One way around this is the “divide and conquer” approach [122,123], which is often applied to in vitro studies. For this, e.g., the structure, interaction sites, and/or posttranslational modifications are first determined at high resolution for isolated peptides or domains of a multi-domain protein before interpreting sparse data of the entire system. The latter might be confirmed finally by mass spectroscopy approaches [124]. “Divide and conquer” was also successful when implementing in-cell NMR and cell-lysate NMR. As described in the previous sections, isolated regulatory sites (signaling peptide sequences or isolated domains) were studied in terms of phosphorylation under in-cell/solution NMR conditions. Some examples include histone 3 modification [125], Elk-1 transactivation domain (TAD) [102], HIV SV40 large T antigen [20], N-terminal extensions to the globular protein G B1 domain [126], and N-terminal transactivation domain of human p53 [106].

**Figure 5:**
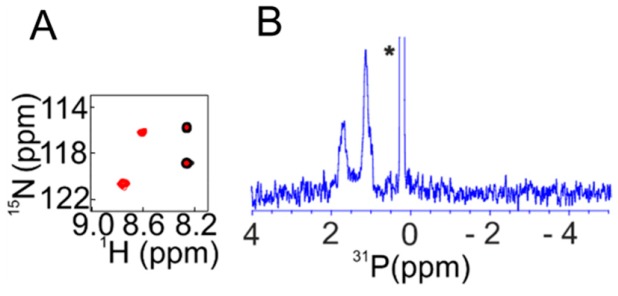
NMR signature of serine phosphorylation using a short peptide (A57-F86 selectively labeled with ^15^N-Ser66 and ^15^N-Ser76) of p19^INK4d^ modified by HeLa cell lysate. (**A**) Overlaid 2D ^1^H–^15^N HSQC spectra of untreated (black) and treated (red) peptide showing the typical low-field shift of the serine amide proton upon side-chain phosphorylation. (**B**) ^31^P NMR spectra of the phosphorylated A57-F86 peptide. The asterisk in (**B**) shows the signal of the phosphate buffer.

While studying the conformational changes of p19^INK4d^ (see previous section), the following question arose: why is the phosphorylation of Ser66 required first before Ser76 can be modified by a second kinase? When modifying a short peptide containing both serine residues by cell lysates, this sequential process was not observed (Figure 5). It turned out that Ser76 is well buried in AR3 of p19^INK4d^ and that the corresponding kinase CDK1 typically phosphorylates regulatory sites in exposed loop structures [24,127]. The role of the phosphate group at Ser66 is to destabilize p19^INK4d^ by repulsive interaction with the negative charge of the net dipole moments of helices 4 and 6 of AR2 and AR3. This could be shown by NMR-detected hydrogen–deuterium exchange of the backbone amides [24,128]. This thermodynamic destabilization allowed CDK1 to reach and modify Ser76. These examples substantiate that “divide and conquer” is not only a feasible approach for in vitro structural biology, but also for in-cell NMR applications. 

## 7. Outlook of In-Cell NMR

In this review, the advances of traditional NMR virtues (structure and dynamics of proteins and hyperpolarization of small organic compounds) toward in-cell applications deciphering protein–small molecule interactions, metabolic pathways, and protein phosphorylation were summarized. The field moved from proof-of-principle to established protocols for a broad range of applications, to gain insight into the physiological and pathological processes of living cells. Despite the huge number of crystal structures in the protein databank and the “resolution revolution” in cryo-electron microscopy, one has to keep in mind that lattice-embedded proteins or vitrified biomacromolecules at very low temperatures may not reveal all of the molecule’s functional properties. In-cell NMR can bridge, to some extent, these discrepancies, and further breakthroughs in the field are to be expected, especially through interdisciplinary studies, to derive general principles to tackle the functions of cellular systems and new avenues of biological research. The remaining challenges involve those regarding low concentrations and resolution. Many improving approaches were summarized using the hyperpolarization of small cellular molecules and isotope-labeled macromolecules. The combination of both improvements allowed the hyperpolarization of proteins in cell lysates [129] and of membrane proteins in living cells using solid-state NMR spectroscopy [130].

## Figures and Tables

**Table 1:** Selection of endogenous target molecules used as reporter probes for hyperpolarization-assisted in-cell NMR studies of metabolic processes, together with their associated field(s) of application.

Target Molecule/Probe	Field of Application (FOA)/Observable
Pyruvate	Pyruvate metabolism; cell permeability; cell lysis; drug efficacy; enzyme activity and reaction fluxes; intracellular pH determination; oncogene signaling; indication of aerobic glycolysis; tricarboxylic acid (TCA) pathway activity; mono carboxylate transporter level/activity; tumor grading
Fumarate	Fumarate metabolism; cell permeability; cell lysis; drug efficacy
Lactate	Enzyme activity and reaction fluxes; tumor grading
Alanine	Enzyme activity and reaction fluxes; enzyme mechanistic studies; tumor grading
Glucose	Gene expression/loss; glycolysis pathway activity; sulfite cytotoxicity; glucose transporter level/activity
Acetate	Enzyme activity and reaction fluxes; intracellular pH determination
Glutamine	Enzyme activity and reaction fluxes
Fructose	Enzyme mechanistic studies
